# Artificial intelligence analysis of continuous wave Doppler spectra to detect reduced left ventricular ejection fraction

**DOI:** 10.1093/ehjdh/ztag137

**Published:** 2026-08-21

**Authors:** Edyta Kaczmarska-Dyrda, Karol A Sadowski, Damian Waląg, Malwina Gonet, Piotr Trochimiuk, Piotr Stankiewicz, Karolina Kryczka, Jacek Kwieciński, Piotr Rudziński, Jacek Kądziela, Michał Kania, Gary S Mintz, Zofia Dzielińska, Marcin Demkow, Hubert Łazarczyk, Łukasz Kalińczuk

**Affiliations:** National Institute of Cardiology, Alpejska 42, 04–628, Warsaw, Poland; National Institute of Cardiology, Alpejska 42, 04–628, Warsaw, Poland; National Institute of Cardiology, Alpejska 42, 04–628, Warsaw, Poland; National Institute of Cardiology, Alpejska 42, 04–628, Warsaw, Poland; National Institute of Cardiology, Alpejska 42, 04–628, Warsaw, Poland; National Institute of Cardiology, Alpejska 42, 04–628, Warsaw, Poland; National Institute of Cardiology, Alpejska 42, 04–628, Warsaw, Poland; National Institute of Cardiology, Alpejska 42, 04–628, Warsaw, Poland; National Institute of Cardiology, Alpejska 42, 04–628, Warsaw, Poland; National Institute of Cardiology, Alpejska 42, 04–628, Warsaw, Poland; National Institute of Cardiology, Alpejska 42, 04–628, Warsaw, Poland; Cardiovascular Research Foundation, New York, USA; National Institute of Cardiology, Alpejska 42, 04–628, Warsaw, Poland; National Institute of Cardiology, Alpejska 42, 04–628, Warsaw, Poland; National Institute of Cardiology, Alpejska 42, 04–628, Warsaw, Poland; National Institute of Cardiology, Alpejska 42, 04–628, Warsaw, Poland

**Keywords:** CW Doppler, AI, LVEF

## Abstract

**Aims:**

To develop an artificial intelligence (AI)-driven approach that analyses continuous-wave (CW) Doppler spectra from the aortic valve (AV) to detect reduced (≤40%) left ventricular ejection fraction (LVEF) without requiring dedicated two-dimensional left ventricular imaging or ECG-gated volumetric analysis for model inference. Current AI solutions for LVEF assessment rely on 2D imaging and ECG signals, limiting utility when data quality is poor.

**Methods and results:**

This retrospective study analysed 4231 aortic CW Doppler recordings from 3988 examinations (3580 patients). Preprocessing yielded 13 359 single-peak images. A CoAtNet-2 neural network was developed using patient-level training and validation cohorts and evaluated on an independent held-out test cohort. The network generated predictions for each single-peak image, and these probabilities were then averaged per examination. Maximum AV blood flow velocity and average CW Doppler pixel intensity were measured. LVEF ≤40% occurred in 20.8% of examinations, associating with lower maximum AV velocity and higher average pixel intensity. In the independent test cohort (782 examinations), the model achieved 85.2% accuracy, 79.0% sensitivity, 86.7% specificity, an AUC of 0.906, an NPV of 94.3%, and a PPV of 59.9%, with robust performance across subgroups.

**Conclusion:**

This proof-of-concept study demonstrates the feasibility of using AI to analyse CW Doppler spectra for rapid, non-invasive identification of reduced LVEF without requiring dedicated two-dimensional left ventricular imaging or ECG-gated volumetric analysis for model inference. By leveraging underutilized echocardiographic Doppler data, this signal-based approach may serve as an adjunctive screening or rule-out tool, particularly when standard imaging or ECG-gated analysis is limited or delayed.

## Graphical Abstract

**Graphical Abstract:**
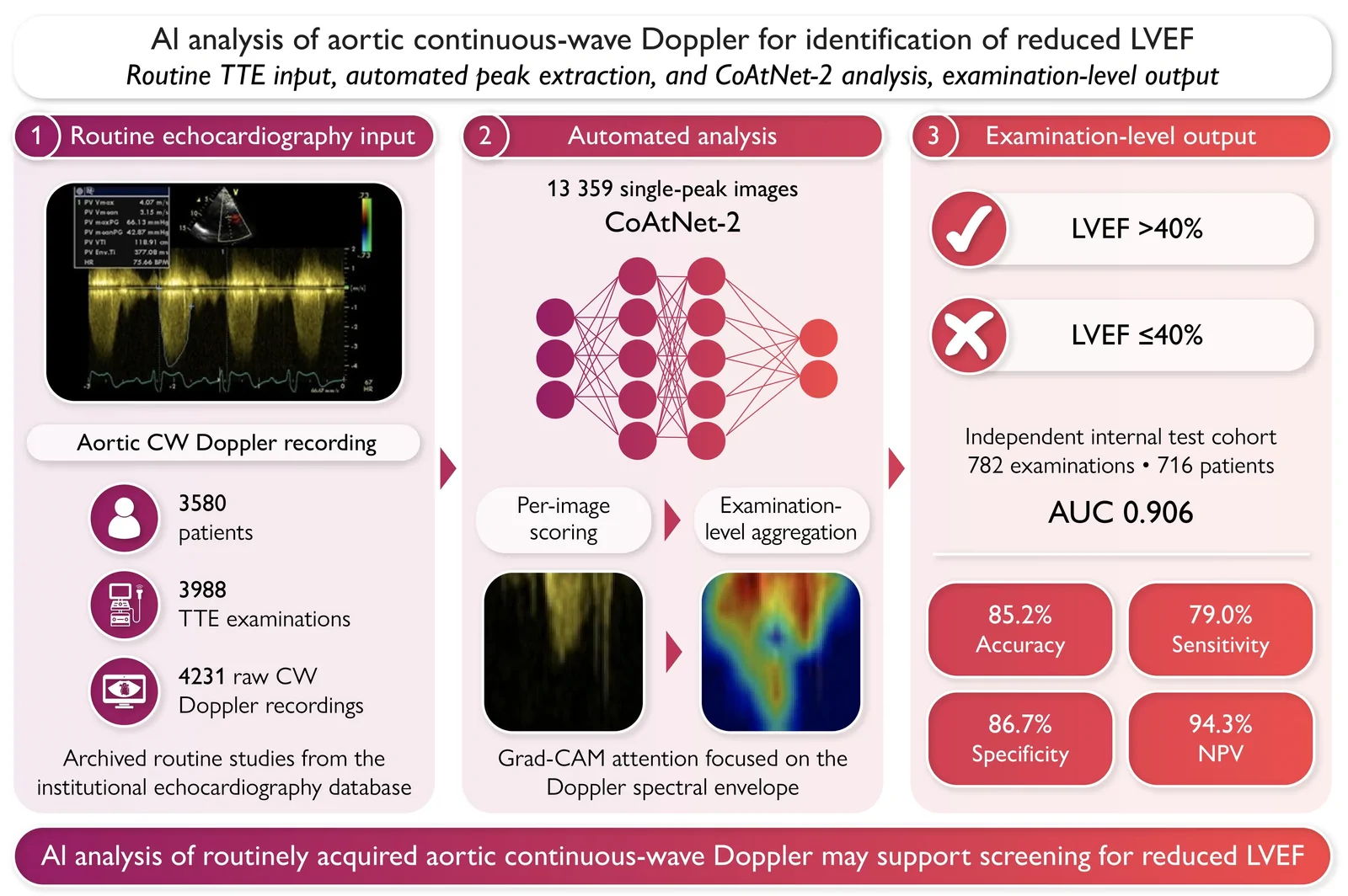

## Introduction

One of the primary diagnostic goals of transthoracic echocardiography (TTE) is to evaluate systolic function of the left ventricle, most often measured by its ejection fraction (LVEF).^1^ Despite well-documented limitations, LVEF remains one of the most frequently measured variables in clinical practice, owing to its well-validated and actionable clinical value.^2^ Accurately assessing LVEF requires technical expertise and experience to recognize coexisting factors that can alter its value. Current methods for determining LVEF rely on TTE imaging of the left ventricular endocardial border at end-systole and corresponding end-diastole, allowing calculation of LVEF as the stroke volume (end-diastolic volume minus end-systolic volume) divided by the end-diastolic volume. These images can be meticulous traced manually or analysed automatically using artificial intelligence (AI)-based tools like GE Healthcare’s ‘AutoEF’ and ‘Caption AI^TM^ AutoEF.’^3,4^ Although such AI-powered solutions can reduce the time and operator variability involved in measuring LVEF, they typically require simultaneous ECG signals and high-quality apical views, which may not be feasible under poor acoustic conditions or stringent time constraints. Meanwhile, every standard TTE exam also collects continuous wave (CW) Doppler data across the aortic valve (AV). In routine practice, only peak and mean velocities are typically measured, leaving the broader spatiotemporal details of the waveform (including details of the curve shape and patterns of the area under the curve) largely underused. Recently, some investigators have begun performing pixel-level analyses of the relevant Doppler spectra—introducing, for instance, a ‘pixel variation score’—to capture more subtle information about flow complexity (in conditions such as mitral regurgitation).^5,6^ We hypothesized that analysing the entire AV CW Doppler envelope—rather than just a handful of peak velocities—could reveal distinctive flow patterns associated with reduced LVEF (≤40%), all without relying on ECG gating or standard imaging views. If successful, this ‘signal-only’ approach may overcome common challenges related to operator expertise, limited imaging conditions, or tight time demands, offering an efficient and robust means to identify depressed systolic function.

In this proof-of-concept study, we propose and evaluate an AI-driven technique that classifies patients solely based on CW Doppler spectra from the AV. We also compare simpler metrics, such as the average pixel intensity of the CW Doppler AV spectra and the maximum blood flow velocity across the AV, to determine their diagnostic value in this context.

## Methods

### Study population

**Figure 1:**
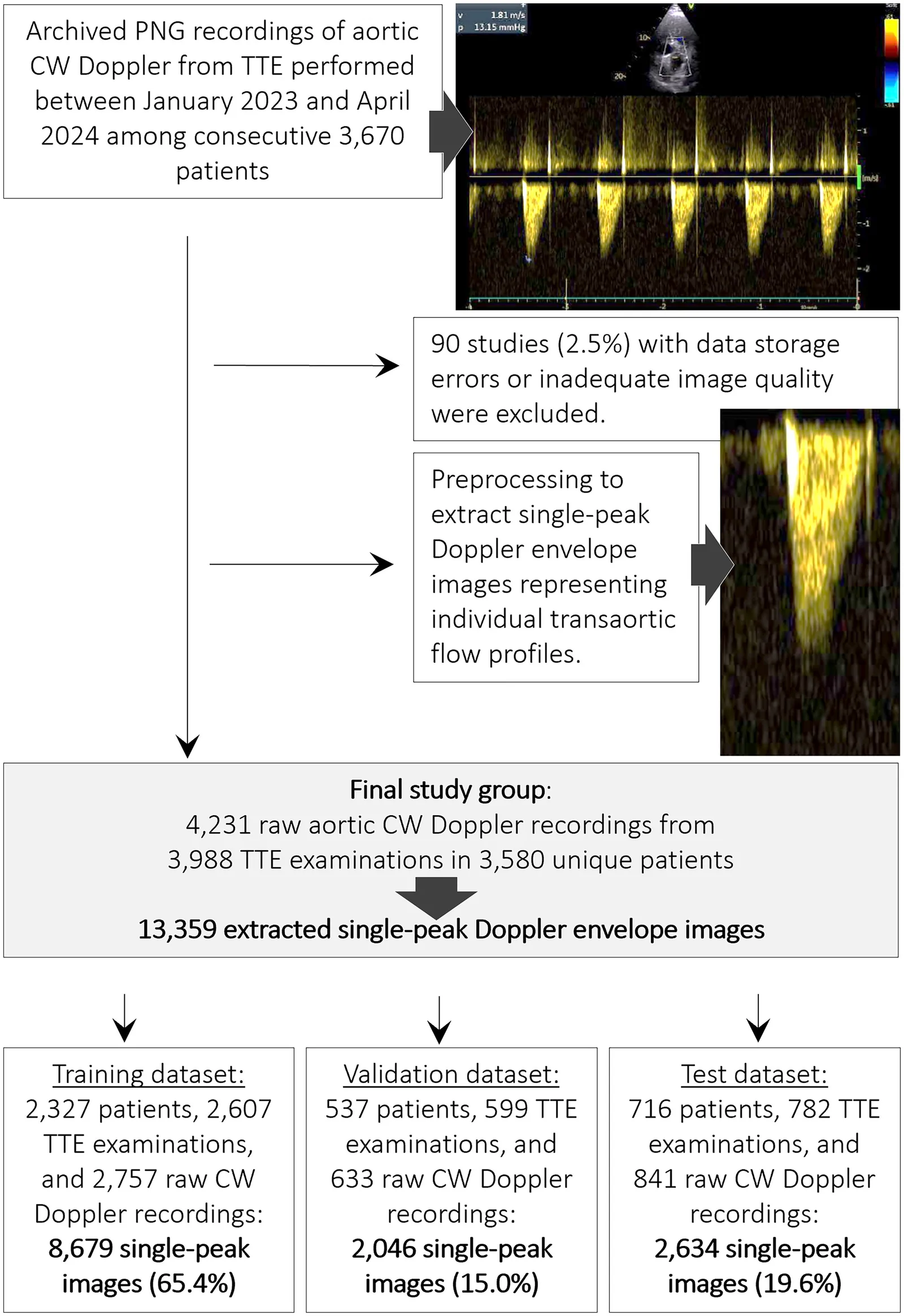
Flowchart showing data collection.

This single-centre, retrospective cohort study was conducted at the National Institute of Cardiology in Warsaw, Poland. All patients there undergo thorough evaluations with advanced diagnostic tools—laboratory tests are run on automated analysers, and TTE examinations are performed by experienced specialists using high-resolution ultrasound systems in accordance with the guidelines of the European Association of Cardiovascular Imaging.^7^ The data generated are high-fidelity and well-suited for diagnostic model development. We screened the institutional archive of transthoracic echocardiographic examinations and corresponding PNG recordings of aortic continuous-wave Doppler spectra. The revised analysis included 4231 raw continuous-wave Doppler recordings from 3988 examinations in 3580 unique patients (*Figure 1*). Patients were eligible if they (1) had detailed baseline comorbidity and clinical data available, and (2) underwent a high-quality CW Doppler assessment of the AV saved as valid PNG images, and (3) had their LVEF measured during the same TTE. Exclusion criteria included severe AV regurgitation or stenosis, congenital heart disease (excluding patent foramen ovale or secundum atrial septal defect), hypertrophic cardiomyopathy with left ventricular outflow tract obstruction, or a prosthetic aortic valve. The study protocol was reviewed and approved by the Ethics Committee of the National Institute of Cardiology, Poland (4.57/VI/24). All procedures complied with the Declaration of Helsinki.

### Echocardiographic assessment

**Figure 2:**
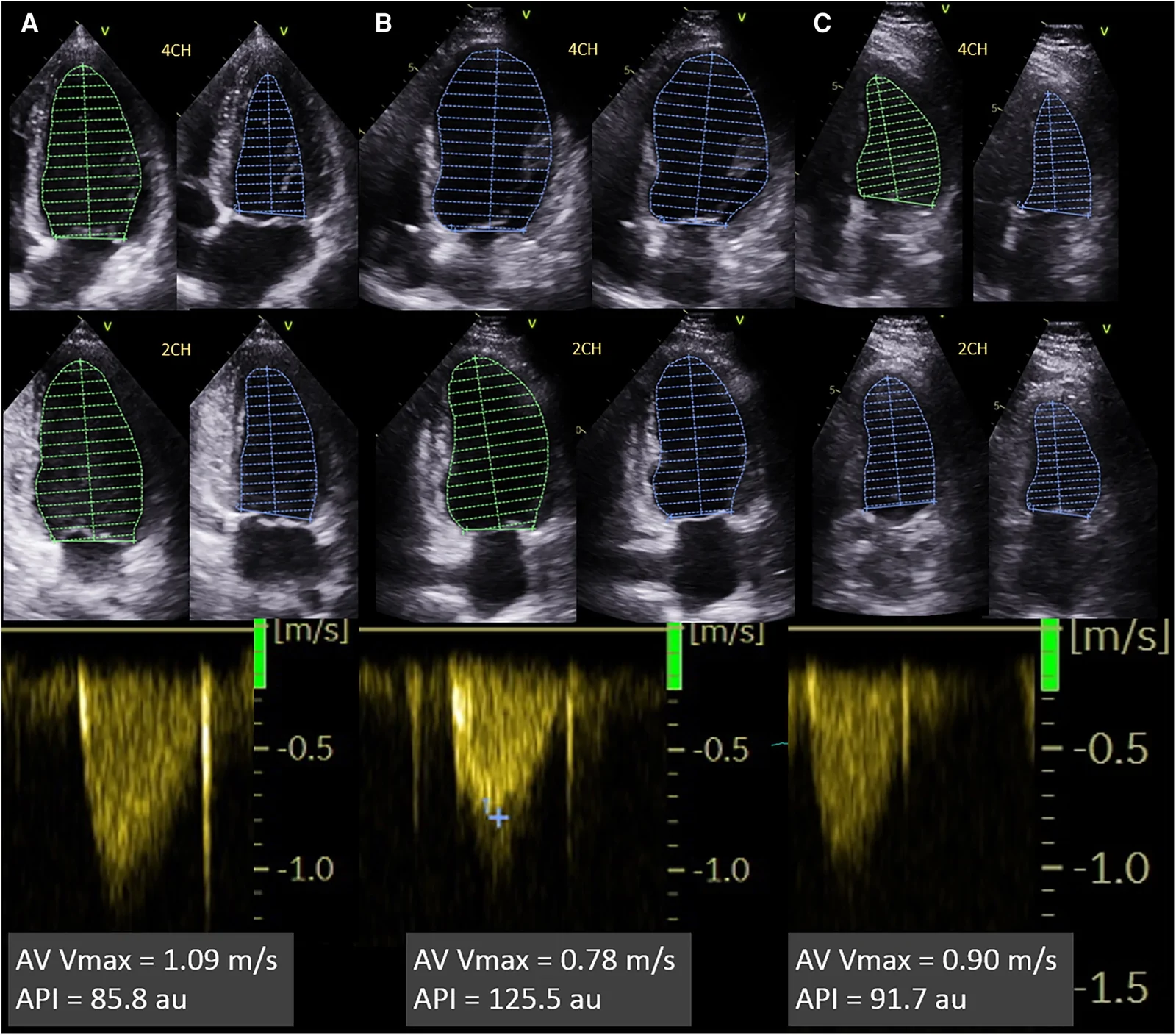
Representative examples of LVEF measurements using the biplane method, combining apical 4- and 2-chamber views at the end-diastole (left column) on end-systole (right column). Panel (*A*) depicts a case with LVEF—64% and normokinesia. Panel (*B*) shows LVEF—17% with antero-latera akinesia, while panel (*C*) illustrates an LVEF—36% with global hypokinesia. Corresponding CW Doppler spectra from the AV, displayed below each panel, include measurements of maximum blood flow velocity (Vmax; m/s) and average pixel intensity (API; in arbitrary units).

Textual reports and the corresponding PNG images were retrieved from the institutional archive. The LVEF was measured using the biplane Simpson’s rule (apical four-chamber and two-chamber views), with no reliance on AI-powered EF measurements (*Figure 2*). End-diastolic and end-systolic diameters of the left ventricle were obtained from the parasternal long-axis view. Interventricular septum and posterior wall thicknesses were measured at end-diastole, and values exceeding 10 mm in men or 9 mm in women were classified as increased. Any segmental abnormality (hypokinesis, akinesis, or dyskinesis) was noted as a regional wall motion abnormality. Left atrial enlargement was defined as an atrial area >20 cm^2^ at end-systole, excluding pulmonary veins and the atrial appendage. Right ventricular dimensions were checked in parasternal or apical views, and enlargement was recorded if measurements surpassed guideline thresholds (e.g. >35 mm in the parasternal outflow tract). Right atrial enlargement was documented when area measurements exceeded 15 cm^2^ in women or 16 cm^2^ in men. Tricuspid annular plane systolic excursion was measured in M-mode at the lateral tricuspid annulus (<17 mm suggesting right ventricular systolic dysfunction). When feasible, the maximum tricuspid regurgitant jet velocity was used to estimate right ventricular systolic pressure, and pulmonary artery acceleration time was recorded if adequate signals were obtained. For valvular assessment, aortic, mitral, and tricuspid regurgitations were classified as mild, moderate, or severe by integrating 2D and colour Doppler data. A bicuspid aortic valve was noted if only two leaflets or a single raphe were visualized. Aortic root size was measured in the parasternal long-axis view, applying standard body surface area-adjusted cut-offs for dilatation. Finally, CW Doppler across the AV was used to record and measure peak blood flow velocities (m/s).

### Average pixel intensity calculation

The average pixel intensity (API) of the entire CW Doppler spectral envelope was measured for each examination. First, uncompressed PNG images of the Doppler spectra were converted to greyscale. Next, the CW Doppler envelope (area of interest) was manually traced in ImageJ software,^8^ capturing the full region of the spectral signal. ImageJ then computed the API by aggregating the intensities of the enclosed pixels and plotting pixel-intensity changes over time. Throughout data acquisition, machine settings that could influence the average pixel intensity—such as CW Doppler output power, transmission frequency, compression, and gain—were standardized and applied identically across examinations. In particular, the gain level was held at 6 dB, ensuring the greyscale histogram remained between 0 arbitrary units (absolute black) and 255 arbitrary units (absolute white) across all spectra. This approach minimized the risk of over- or under-saturation, thus preserving image quality and consistency in the average pixel intensity calculation.

### Data preparation

All data processing and model training were conducted using Python and the PyTorch library, with OpenCV employed for image manipulation. For each CW Doppler PNG file, the Doppler spectrum was first cropped to isolate the region of interest. A peak detection algorithm was then applied to identify individual spectral ‘peaks,’ each encompassing a distinct blood flow velocity envelope over time. Peak extraction was performed with a deterministic multi-step image-processing pipeline that localized the baseline, denoised and thresholded the spectral signal, converted the Doppler envelope into a one-dimensional trace, smoothed it with Akima 1-D interpolation, and identified individual systolic peaks. Each peak-centred snippet plus its immediate surroundings formed a single training image. This preprocessing served only to segment each continuous recording into temporally aligned single-peak images; no manual tracings or derived haemodynamic measurements were used as model inputs. Applying this procedure to the revised dataset yielded 13 359 PNG images of single CW Doppler envelopes from 4231 raw recordings, including 8679 training images from 2607 examinations, 2046 validation images from 599 examinations, and 2634 test images from 782 examinations (*Figure 2*).

### Model development

A CoAtNet-2 hybrid convolution-attention neural network, pre-trained on ImageNet-12k and fine-tuned on ImageNet-1k, was used as the base model. To minimize information leakage, the dataset was split strictly at the patient level into training (2327 patients, 2607 examinations), validation (537 patients, 599 examinations), and independent held-out test (716 patients, 782 examinations) cohorts. The validation subset was used only for hyperparameter tuning, early stopping, and threshold selection; all final performance metrics were calculated on the independent test cohort. To enhance generalization, training images underwent geometric and photometric augmentation, blur or sharpening, Gaussian noise injection, and simulated signal dropout, whereas validation and test images were only resized and normalized. The model was trained for up to 50 epochs with AdamW, inverse-frequency class weights, dropout of 0.2, stochastic depth of 0.4, bfloat16 precision, and a fixed random seed. For the first 10 epochs the pre-trained backbone was frozen, after which the full network was unfrozen and optimized using layer-wise learning-rate decay, a 5-epoch linear warm-up, cosine annealing, weight decay of 0.07, label smoothing of 0.05, exponential moving average monitoring (decay 0.9995), and early stopping based on validation AUC. All experiments were performed in Python 3.13.7 using PyTorch 2.8.0, timm 1.0.20, Albumentations 2.0.8, scikit-learn 1.7.2, Matplotlib 3.10.6, and grad-cam 1.5.5 on a workstation equipped with an NVIDIA RTX 3500 Ada Generation GPU.

Because each examination could have multiple single-peak CW Doppler images, an examination-level aggregation step was applied. The model generated a probability for each individual peak image, and these probabilities were averaged across all peaks from the same examination to obtain the final examination-level score. If the mean examination-level probability met the threshold for reduced LVEF (≤40%), the examination was classified as LVEF ≤40%; otherwise, it was classified as LVEF >40%. On average, each examination-level score in the test set was derived from 3.37 ± 1.31 individual peak images. For interpretability, Gradient-weighted Class Activation Mapping (Grad-CAM) was applied to representative correctly classified single-peak test images to generate class-discriminative heat maps highlighting the regions that contributed most strongly to the model’s prediction.

### Statistical analysis

Categorical variables are reported as counts (n) and percentages and were compared using Pearson’s χ^2^ or Fisher’s exact tests, as appropriate. Continuous variables were first checked for normality with the one-sample Kolmogorov-Smirnov test. Normally distributed data are expressed as means ± standard deviation (SD) and compared with Student’s t or one-way ANOVA tests. Non-normally distributed variables are reported as medians with interquartile ranges (IQRs), with group differences assessed using the Mann–Whitney U or Kruskal-Wallis tests. Zero values in the spreadsheet were treated as missing for body mass index, left ventricular dimensions, right ventricular systolic pressure, tricuspid annular plane systolic excursion, pulmonary artery acceleration time, and maximal aortic-valve flow velocity. Model performance for identifying LVEF ≤40% was evaluated at both the per-examination and per-image levels using receiver-operating-characteristic (ROC) analysis. The optimal decision threshold was determined on the validation set by maximizing Youden’s J index, yielding fixed thresholds of 0.4596 for per-examination analyses and 0.5707 for per-image analyses. We report area under the ROC curve (AUC), accuracy, sensitivity, specificity, positive predictive value (PPV), negative predictive value (NPV), and F1 score, with 95% confidence intervals (CI) derived from 10 000 bootstrap iterations. Data were analysed with PASW Statistics 18 (IBM, Armonk, NY, USA) and Python’s scikit-learn library. Prespecified examination-level subgroup analyses were performed according to sex, age (>66 vs. ≤66 years), median left ventricular end-diastolic diameter, and the presence or absence of regional left ventricular wall motion abnormality. For the Grad-CAM figure, p denotes the per-image predicted probability of LVEF ≤40%; the per-image threshold is displayed as 0.57 after rounding from 0.5707.

## Results

### Demographics and baseline clinical data

The study cohort included 3580 unique patients contributing 3988 examinations (40.8% female), with a median age of 62.0 years (interquartile range [IQR] 45.0–71.0) (*Table 1*). Hypertension was present in 62.1%, dyslipidaemia in 10.5%, and diabetes mellitus in 16.4%. A prior stroke or transient ischaemic attack was reported by 16.5%, and 3.8% were diagnosed with chronic kidney disease. Hypertrophic, dilated or ischaemic cardiomyopathy was identified in 8.8%, and 5.5% had an implanted cardiac device (pacemaker or implantable cardioverter-defibrillator). Coronary revascularization—whether percutaneous or surgical—had been performed in 14.7% of examinations, and 19.2% had experienced a prior myocardial infarction. Atrial fibrillation (paroxysmal or persistent) was found in 20.2%, while 3.6% had bundle branch block. Overall, 828 examinations (20.8%) had LVEF ≤40%.

**Table 1:** Baseline demographic and clinical characteristics of the examination-level cohort (3580 unique patients with 3988 examinations)

	LVEF ≤40% (n = 828; 20.8%)	LVEF >40% (n = 3160; 79.2%)	Training dataset (n = 2607; 65.4%)	Validation dataset (n = 599; 15.0%)	Test dataset (n = 782; 19.6%)	*P*-value^a^	*P*-value^b^
Age, years	65.0 (56.0–73.0)	61.0 (43.0–70.0)	62.0 (45.5–71.0)	60.0 (45.0–70.0)	61.5 (44.2–71.0)	<0.001	0.305
Female, n (%)	174 (21.0)	1453 (46.0)	1029 (39.5)	269 (44.9)	329 (42.1)	<0.001	0.037
Body mass index	29.0 (25.8–32.3)	27.7 (24.9–30.9)	27.9 (25.1–31.2)	27.7 (24.7–31.3)	28.4 (25.5–31.3)	<0.001	0.453
Hypertension, n (%)	559 (67.5)	1920 (60.8)	1641 (62.9)	349 (58.3)	489 (62.5)	<0.001	0.100
Dyslipidemia, n (%)	106 (12.8)	311 (9.8)	283 (10.9)	55 (9.2)	79 (10.1)	0.013	0.452
Diabetes mellitus, n (%)	203 (24.5)	453 (14.3)	433 (16.6)	84 (14.0)	139 (17.8)	<0.001	0.164
Chronic kidney disease, n (%)	57 (6.9)	93 (2.9)	90 (3.5)	23 (3.8)	37 (4.7)	<0.001	0.255
Hypertrophic, dilated or ischaemic cardiomyopathy, n (%)	161 (19.4)	190 (6.0)	226 (8.7)	61 (10.2)	64 (8.2)	<0.001	0.396
Prior myocardial infarction, n (%)	348 (42.0)	416 (13.2)	506 (19.4)	107 (17.9)	151 (19.3)	<0.001	0.682
Prior coronary angioplasty, n (%)	173 (20.9)	298 (9.4)	316 (12.1)	59 (9.8)	96 (12.3)	<0.001	0.270
Prior coronary artery bypass grafting, n (%)	83 (10.0)	87 (2.8)	114 (4.4)	20 (3.3)	36 (4.6)	<0.001	0.460
Prior stroke or transient ischaemic attack, n (%)	237 (28.6)	420 (13.3)	429 (16.5)	105 (17.5)	123 (15.7)	<0.001	0.670
Atrial fibrillation, n (%)	273 (33.0)	531 (16.8)	514 (19.7)	112 (18.7)	178 (22.8)	<0.001	0.111
Bundle branch block (left/right), n (%)	64 (7.7)	80 (2.5)	108 (4.1)	16 (2.7)	20 (2.6)	<0.001	0.047
Pacemaker or implantable cardioverter defibrillator, n (%)	125 (15.1)	93 (2.9)	148 (5.7)	27 (4.5)	43 (5.5)	<0.001	0.524

^a^Comparison between LVEF ≤40% and LVEF >40%.

^b^Comparison across the training, validation, and test datasets.

Continuous variables are presented as median (interquartile range); *P*-values were calculated with Mann–Whitney U or Kruskal–Wallis tests.

Categorical variables are presented as n (%); *P*-values were calculated with χ^2^ tests or Fisher’s exact tests, as appropriate.

Percentages are based on the number of examinations in each column.

For body mass index, LV dimensions, RVSP, TAPSE, pulmonary artery acceleration time, and AV Vmax, zero values in the spreadsheet were treated as missing.

### Detailed echocardiographic data

Comparative echocardiographic data for those with LVEF ≤40% vs. LVEF >40% have been presented in *Table 2*. Patients with LVEF ≤40% had significantly larger left ventricular end-diastolic and end-systolic diameters [61.0 (56.0–67.0) vs. 49.0 (45.0–53.0) mm and 49.0 (42.0–57.0) vs. 30.0 (26.0–35.0) mm, respectively; both *P* < 0.001]. These changes were accompanied by elevated intracardiac pressures, decreased tricuspid annular plane systolic excursion, and reduced pulmonary artery acceleration times in the LVEF ≤40% subgroup (all *P* < 0.001).

**Table 2:** Echocardiographic characteristics of the examination-level cohort (3580 unique patients and 3988 examinations)

	LVEF ≤40% (n = 828; 20.8%)	LVEF >40% (n = 3160; 79.2%)	Training dataset (n = 2607; 65.4%)	Validation dataset (n = 599; 15.0%)	Test dataset (n = 782; 19.6%)	*P*-value^a^	*P*-value^b^
**Left ventricle**							
End-diastolic diameter, mm	61.0 (56.0–67.0)	49.0 (45.0–53.0)	50.0 (46.0–56.0)	50.6 (46.0–57.0)	51.0 (46.0–56.0)	<0.001	0.858
End-systolic diameter, mm	49.0 (42.0–57.0)	30.0 (26.0–35.0)	33.0 (27.0–40.0)	33.0 (27.0–40.0)	33.0 (27.0–40.0)	<0.001	0.666
EF, %	32.0 (25.0–38.2)	60.0 (55.0–65.0)	60.0 (45.0–65.0)	60.0 (45.0–65.0)	60.0 (48.2–65.0)	<0.001	0.087
**Right ventricle**							
Systolic pressure, mmHg	40.0 (31.0–50.0)	30.0 (26.0–36.0)	32.0 (27.0–40.0)	31.0 (27.5–40.0)	32.0 (26.0–40.0)	<0.001	0.707
Tricuspid annular plane systolic excursion, mm	18.0 (15.0–21.0)	22.0 (19.0–25.0)	21.0 (18.0–25.0)	21.0 (18.0–24.0)	22.0 (18.0–25.0)	<0.001	0.367
Pulmonary artery acceleration time, ms	91.0 (74.0–111.0)	114.0 (96.0–133.0)	107.0 (89.0–129.0)	110.0 (94.0–133.0)	111.0 (94.8–133.0)	<0.001	0.060
**Valvular evaluation**							
Maximal AV blood flow velocity, m/s	1.29 (1.10–1.50)	1.40 (1.20–1.70)	1.40 (1.20–1.70)	1.40 (1.20–1.60)	1.40 (1.20–1.60)	<0.001	0.810
Average AV CW Doppler spectrum pixel intensity, au	170.04 (144.60–196.07)	165.37 (142.09–192.69)	166.40 (143.11–193.54)	165.08 (141.41–190.61)	166.27 (141.81–194.35)	0.009	0.489
Mild aortic regurgitation, n (%)	167 (20.2)	597 (18.9)	517 (19.8)	99 (16.5)	148 (18.9)	0.406	0.177
Moderate aortic regurgitation, n (%)	42 (5.1)	113 (3.6)	92 (3.5)	30 (5.0)	33 (4.2)	0.047	0.208
Moderate/severe mitral regurgitation, n (%)	378 (45.7)	364 (11.5)	486 (18.6)	106 (17.7)	150 (19.2)	<0.001	0.779
Moderate/severe tricuspid regurgitation, n (%)	201 (24.3)	339 (10.7)	365 (14.0)	83 (13.9)	92 (11.8)	<0.001	0.269

^a^Comparison between LVEF ≤40% and LVEF >40%.

^b^Comparison across the training, validation, and test datasets.

Continuous variables are presented as median (interquartile range); *P*-values were calculated with Mann–Whitney U or Kruskal–Wallis tests.

Categorical variables are presented as n (%); *P*-values were calculated with χ^2^ tests or Fisher’s exact tests, as appropriate.

Percentages are based on the number of examinations in each column.

For body mass index, LV dimensions, RVSP, TAPSE, pulmonary artery acceleration time, and AV Vmax, zero values in the spreadsheet were treated as missing.

Regurgitation grades were harmonized from mixed numeric and text entries in the master spreadsheet.

Mild aortic regurgitation denotes mild regurgitation only; moderate aortic regurgitation denotes moderate regurgitation only; moderate/severe mitral and tricuspid regurgitation denote moderate or greater regurgitation.

Average AV CW Doppler spectrum pixel intensity was not available as a row-level variable in the master spreadsheet and is therefore not shown.

While maximum AV blood flow velocity was smaller and average CW Doppler pixel intensity was greater in examinations with LVEF ≤40% [1.29 (1.10–1.50) vs. 1.40 (1.20–1.70) m/s, *P* < 0.001; and 170.04 (144.60–196.07) vs. 165.37 (142.09–192.69) arbitrary units, *P* = 0.009, respectively], moderate aortic regurgitation and moderate or severe tricuspid and mitral regurgitations were more common among the LVEF ≤40% group, whereas mild aortic regurgitation did not differ significantly between groups (*Table 2*).

### Model results

**Figure 3:**
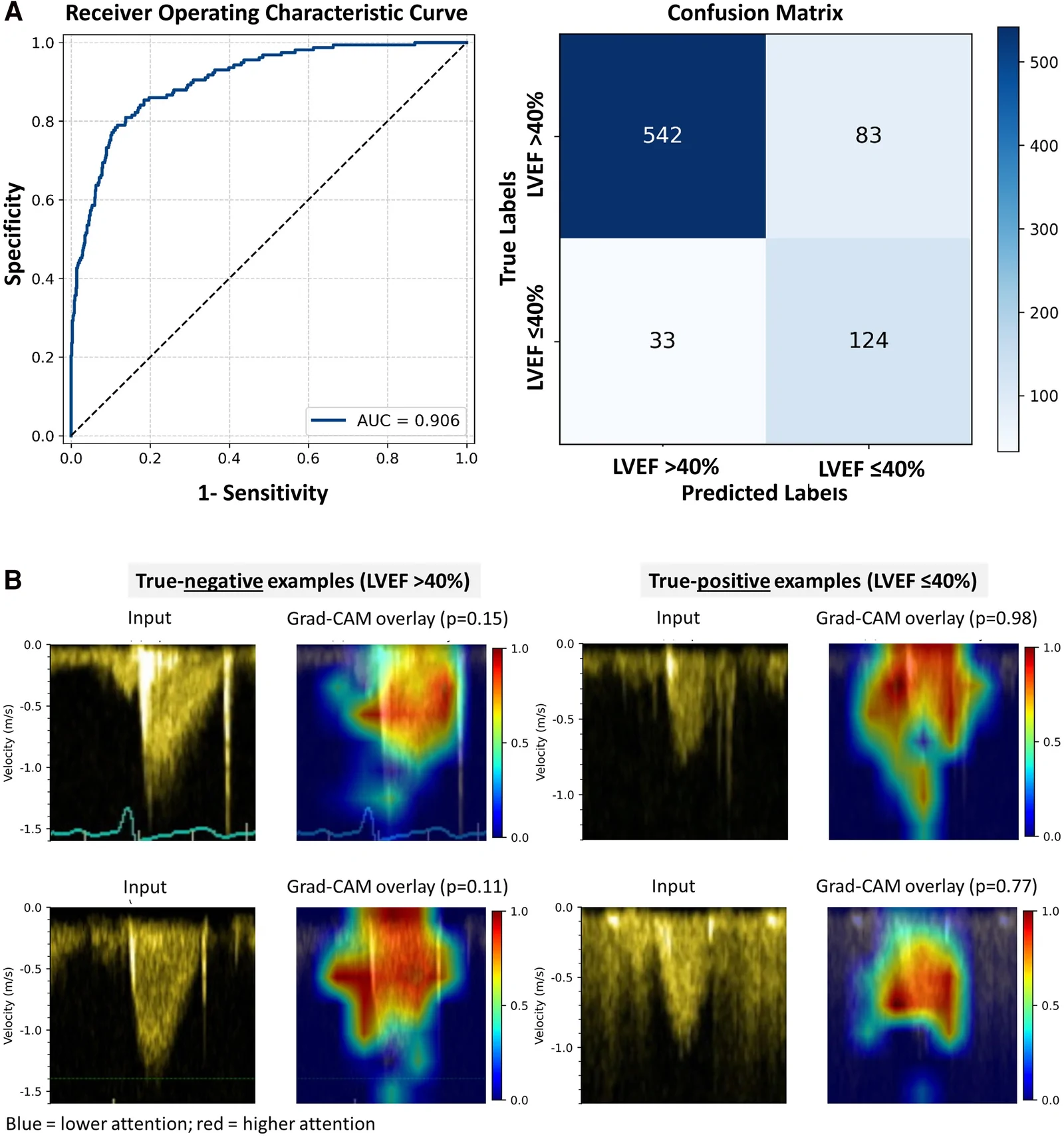
Examination-level performance and Grad-CAM interpretability of the final AI model for identifying LVEF ≤40%. (*A*) Receiver-operating characteristic curve and confusion matrix for the independent examination-level test cohort. Final examination-level predictions were evaluated using a fixed threshold of 0.4596. (*B*) Representative correctly classified test-set single-peak aortic continuous-wave Doppler images and corresponding Grad-CAM overlays. The left subpanel shows true-negative examples (LVEF >40%), and the right subpanel shows true-positive examples (LVEF ≤40%). Warmer colours indicate regions contributing more strongly to the model’s decision, predominantly along the Doppler spectral envelope and peak contour. In panel (*B*), p denotes the per-image predicted probability of LVEF ≤40%; the per-image classification threshold was 0.5707 (rounded to 0.57 in the figure).

A total of 4231 raw aortic continuous-wave Doppler recordings from 3988 TTE examinations in 3580 unique patients, yielding 13 359 extracted single-peak images after preprocessing were analysed (*Figure 1*). Following data preprocessing and model development, the final AI classifier achieved an accuracy of 85.2% (95% CI: 82.6–87.6), sensitivity of 79.0% (95% CI: 72.5–85.1), specificity of 86.7% (95% CI: 84.0–89.3), F1 score of 68.1% (95% CI: 62.4–73.4), PPV of 59.9% (95% CI: 52.9–66.7), NPV of 94.3% (95% CI: 92.3–96.1), and an AUC of 0.906 (95% CI: 0.879–0.930) (*Figure 3A*). The corresponding confusion matrix comprised 542 true negatives, 124 true positives, 83 false positives, and 33 false negatives. Grad-CAM analysis of representative correctly classified single-peak test images showed that model attention was concentrated predominantly along the Doppler spectral envelope and peak contour, with minimal activation in the background (*Figure 3B*). Subgroup analyses showed robust performance across prespecified demographic and echocardiographic strata. For instance, AUC values ranged from 0.832 to 0.930 when stratified by age, left ventricular size, sex, and presence or absence of regional wall motion abnormalities (see Supplementary material online, *Table S3*). Discrimination and sensitivity were similar in women and men (AUC 0.910 vs. 0.901; sensitivity 78.8% vs. 79.0%), whereas the positive predictive value was lower in women (40.0% vs. 69.0%). Comprehensive subgroup metrics appear in Supplementary material online, *Table S3*.

## Discussion

In this proof-of-concept study, we developed and validated a deep learning model that uses CW Doppler signals from the AV to identify a markedly reduced LVEF (≤40%). Although our dataset was single-centre and relatively small, the final examination-level model achieved an AUC of 0.906, accuracy of 85.2%, sensitivity of 79.0%, specificity of 86.7%, PPV of 59.9%, and NPV of 94.3% on the independent held-out test cohort. These findings support the feasibility of a signal-based approach that does not require dedicated two-dimensional LV imaging or ECG-gated volumetric analysis for model inference. The performance profile, particularly the high NPV, is most compatible with an adjunctive rule-out or triage use case rather than a stand-alone replacement for formal echocardiographic quantification of LVEF.

### Comparison with existing AI solutions

Our Doppler-based AI model compares favourably with the latest methods, such as GE Healthcare’s ‘Caption AI^TM^ AutoEF,’ which reports an accuracy of 92%, an NPV of 96%, and a PPV of 83% for the automated detection of LVEF ≤35%, with a sensitivity of 77% and specificity of 79% in identifying patients with reduced LVEF from single views in a point-of-care setting.^9,10^ However, direct comparison should be interpreted cautiously because the target LVEF threshold, input modality, dataset size, and clinical setting differ from those of the present study. This algorithm was trained on a database of over 50 000 echocardiograms, including multiple apical 2- and 4-chamber views, collected over a 10-year period at a single US centre.^9^ Importantly, those solutions remain heavily dependent on high-quality apical views and ECG signal synchronization.^11^ In contrast, our technique uses continuous-wave Doppler data acquired during routine echocardiography and does not require dedicated two-dimensional LV imaging or ECG-gated volumetric analysis for model inference. This signal-based design may be advantageous when apical imaging is delayed, limited, or suboptimal, although positive outputs still require standard echocardiographic confirmation.

### Subgroup analyses and pixel intensity

Examination-level discrimination remained acceptable across the prespecified subgroups, with AUCs of 0.910 in women, 0.901 in men, 0.926 in patients older than 66 years, 0.891 in those aged 66 years or younger, 0.885 in examinations with > median left ventricular end-diastolic diameter, 0.832 in those with ≤ median diameter, 0.861 in examinations with regional wall motion abnormalities, and 0.930 in those without them. These findings suggest that the Doppler signal retains clinically relevant information across diverse demographic and echocardiographic contexts, although sensitivity and PPV varied according to subgroup composition and prevalence. Sensitivity was similar in women and men (78.8% vs. 79.0%), whereas the lower PPV in women (40.0% vs. 69.0%) is likely explained at least in part by the lower prevalence of LVEF ≤40% in women rather than by weaker model discrimination.^12,13^ Although maximum blood flow velocity and average pixel intensity both differed between patients with reduced vs. preserved LVEF, each measure alone proved insufficient to identify severely lowered LVEF. Instead, the network seems to leverage nuanced temporal and shape-based features of the Doppler envelope that go beyond simple brightness or peak identification.^14^ This interpretation is supported qualitatively by Grad-CAM heat maps, in which attention concentrated on the Doppler spectral envelope and peak contour with minimal activation in background regions.

An additional methodological nuance is that the network analysed cropped Doppler spectrum images saved without explicit x- and y-axis information (time and velocity in m/s). Classical Doppler studies have shown that derived indices such as peak aortic acceleration can correlate strongly with global left ventricular systolic function,^15,16^ yet in our cohort maximum AV velocity and average pixel intensity were only weak standalone predictors of LVEF ≤40%. Together, these observations argue against the model merely reproducing a single calibrated Doppler measure or analytically summing the envelope area and instead support the interpretation that it learned higher-order morphological features of the spectrum image itself.

### Relevance of other Doppler envelope patterns

While we focused on detecting decreased LVEF from AV Doppler data, echocardiographic Doppler signals can also reveal numerous distinctive patterns associated with specific conditions—for example, parvus et tardus in high-grade aortic stenosis, dagger-shaped outflow in hypertrophic obstructive cardiomyopathy, and the cut-off sign in acute severe mitral regurgitation.^17^ These patterns illustrate the breadth of diagnostic information embedded in Doppler waveforms. Future AI-based systems could integrate pixel-level and shape-based analyses to assess both systolic/diastolic functions and a range of valvular disorders in one streamlined approach.^18–20^

### Clinical implications and future directions

By capitalizing on commonly collected but underutilized CW Doppler data, our method could fit into routine workflows as an adjunctive screening or rule-out tool in time-critical scenarios or when dedicated LV imaging or ECG-gated analysis is not immediately available or is suboptimal. A positive model output should not be interpreted as a stand-alone diagnosis of reduced LVEF and should instead trigger comprehensive echocardiographic assessment. Beyond ejection fraction estimation, related AI architectures may eventually be extended to other valvular or haemodynamic Doppler signatures.^14^ Ongoing efforts should prioritize external prospective multicentre validation, calibration analyses, and testing across vendors and acquisition settings before any clinical deployment.

### Limitations

A key limitation of our study is that, despite the cohort of 3580 unique patients and 3988 examinations, it remains a single-centre retrospective analysis with internal validation only. This limits inference about performance across other centres, vendors, acquisition settings, and patient populations, and external prospective multicentre validation is required before clinical deployment. In addition, we excluded cases with poor-quality Doppler signals, potentially biasing the findings towards higher-fidelity data. Certain Doppler flow parameters—such as mean blood flow velocity through the aortic valve and the velocity-time integral of aortic flow—were not available, limiting further insight into the model’s underlying mechanisms. We also did not perform a blinded agreement study against manual envelope tracings, because the model was trained on raw pixel data from cropped single-peak images rather than on manually or automatically traced envelopes or tracing-derived measurements. Final performance reporting was based on held-out examination-level predictions after patient-level splitting to prevent leakage; repeated examinations and longitudinal changes in LVEF were not evaluated, and no formal reproducibility analysis was performed. Another limitation is that the Doppler spectra were not labelled with corresponding velocity scales and acquisition shifts in this model iteration because of design choices during data preparation. Although performance remained strong without these metadata, this should not be interpreted as evidence of broad generalizability. Future studies could address these limitations by incorporating these additional parameters in larger and more diverse datasets and by examining whether the AI model consistently predicts changes in LVEF across multiple examinations or over time.

## Conclusion

In summary, this deep learning model based solely on CW Doppler spectra shows diagnostic accuracy comparable to some well-known imaging-based AI tools. Its strengths include high NPV, lack of dependency on ECG gating or 2D image quality, and sustained efficacy across multiple subgroups. While there remain avenues for improvement—particularly in increasing PPV and broader validation—this study provides a foundation for signal-only echocardiographic AI and points to future possibilities for more continuous, quick, and resource-efficient cardiac assessments in clinical practice.

## Supplementary Material

ztag137_Supplementary_Data

## Data Availability

The data underlying this article cannot be shared publicly due to patient privacy considerations and institutional data governance requirements. The dataset is currently being used in ongoing research aimed at further development and validation of the algorithm. De-identified data may be shared upon reasonable request to the corresponding author, subject to approval by the National Institute of Cardiology and applicable legal and institutional governance procedures.

## Relationships with industry and other entities

The authors have reported that they have no relationships relevant to the contents of this paper to disclose.
