# Supplementary material for: Artificial intelligence analysis of continuous wave Doppler spectra to detect reduced left ventricular ejection fraction
Source: Eur Heart J Digit Health. 2026 Aug 21;7(8):ztag137. doi: 10.1093/ehjdh/ztag137 (PMC13550941; doi:10.1093/ehjdh/ztag137)
Supplement: Supplementary file 1 [file ztag137_supplementary_data.docx]

**Supplementary Table 3**. Examination-level AI model performance for diagnosis of LVEF ≤40% in the independent test cohort and prespecified demographic and echocardiographic subgroups

| **Subgroup** | **AUC** | **Accuracy, %** | **Sensitivity, %** | **Specificity, %** | **Positive predictive value, %** | **Negative predictive value, %** | **F1 score** |
| --- | --- | --- | --- | --- | --- | --- | --- |
| Female (n=329) | 0.910 (0.861 - 0.952) | 86.0 (82.1 - 89.7) | 78.8 (64.3 - 92.0) | 86.8 (82.8 - 90.5) | 40.0 (28.4 - 52.0) | 97.3 (95.3 - 99.2) | 53.1 (40.4 - 64.1) |
| Male (n=453) | 0.901 (0.866 - 0.932) | 84.5 (81.0 - 87.9) | 79.0 (71.6 - 86.1) | 86.6 (82.8 - 90.2) | 69.0 (61.2 - 76.6) | 91.6 (88.4 - 94.6) | 73.7 (67.4 - 79.4) |
| Age > 66 years (n=283) | 0.926 (0.887 - 0.961) | 85.9 (82.0 - 89.8) | 87.5 (79.0 - 95.3) | 85.4 (80.6 - 89.9) | 63.6 (53.7 - 73.6) | 95.9 (92.9 - 98.5) | 73.7 (65.3 - 81.0) |
| Age <= 66 years (n=499) | 0.891 (0.854 - 0.925) | 84.8 (81.6 - 87.8) | 73.1 (63.7 - 81.9) | 87.4 (84.1 - 90.6) | 57.1 (48.1 - 66.0) | 93.4 (90.8 - 95.8) | 64.2 (56.0 - 71.2) |
| > Median left ventricular end-diastolic diameter (n=414) | 0.885 (0.849 - 0.917) | 81.4 (77.5 - 85.0) | 82.4 (75.9 - 88.4) | 80.9 (76.2 - 85.4) | 69.2 (62.3 - 76.1) | 89.8 (85.8 - 93.4) | 75.2 (69.6 - 80.4) |
| <= Median left ventricular end-diastolic diameter (n=368) | 0.832 (0.710 - 0.925) | 89.4 (86.1 - 92.4) | 46.7 (21.4 - 73.3) | 91.2 (88.2 - 94.1) | 18.4 (6.9 - 31.7) | 97.6 (95.8 - 99.1) | 26.4 (10.5 - 41.9) |
| Regional left ventricular wall motion abnormality (n=280) | 0.861 (0.817 - 0.901) | 78.6 (73.9 - 83.2) | 78.4 (71.3 - 85.1) | 78.8 (71.9 - 85.3) | 77.2 (70.0 - 84.2) | 79.9 (73.2 - 86.2) | 77.8 (72.1 - 82.9) |
| No regional left ventricular wall motion abnormality (n=502) | 0.930 (0.873 - 0.974) | 88.8 (86.1 - 91.6) | 82.6 (65.4 - 96.0) | 89.1 (86.2 - 91.9) | 26.8 (16.7 - 37.7) | 99.1 (98.1 - 99.8) | 40.4 (27.0 - 52.6) |
| All (n=782) | 0.906 (0.879 - 0.930) | 85.2 (82.6 - 87.6) | 79.0 (72.5 - 85.1) | 86.7 (84.0 - 89.3) | 59.9 (52.9 - 66.7) | 94.3 (92.3 - 96.1) | 68.1 (62.4 - 73.4) |

All metrics were calculated at the per-examination level. Values are presented as point estimates with 95% confidence intervals in parentheses, derived from 10,000 bootstrap iterations. The decision threshold (0.4596) was determined on the validation set using Youden's J index and then fixed for evaluation in the independent test cohort. AUC = area under the receiver operating characteristic curve; LVEF = left ventricular ejection fraction.
